# Neurofilaments contribution in clinic: state of the art

**DOI:** 10.3389/fnagi.2022.1034684

**Published:** 2022-11-01

**Authors:** Constance Delaby, Olivier Bousiges, Damien Bouvier, Catherine Fillée, Anthony Fourier, Etienne Mondésert, Nicolas Nezry, Souheil Omar, Isabelle Quadrio, Benoit Rucheton, Susanna Schraen-Maschke, Vincent van Pesch, Stéphanie Vicca, Sylvain Lehmann, Aurelie Bedel

**Affiliations:** ^1^Université de Montpellier, IRMB, INM, INSERM, CHU de Montpellier, Laboratoire Biochimie-Protéomique clinique, Montpellier, France; ^2^Sant Pau Memory Unit, Hospital de la Santa Creu i Sant Pau—Biomedical Research Institute Sant Pau—Universitat Autònoma de Barcelona, Barcelona, Spain; ^3^Laboratoire de biochimie et biologie moléculaire (LBBM)—Pôle de biologie Hôpital de Hautepierre—CHU de Strasbourg, CNRS, laboratoire ICube UMR 7357 et FMTS (Fédération de Médecine Translationnelle de Strasbourg), équipe IMIS, Strasbourg, France; ^4^Service de Biochimie et Génétique Moléculaire, CHU de Clermont-Ferrand, Clermont-Ferrand, France; ^5^Cliniques universitaires Saint-Luc UCLouvain, Service de Biochimie Médicale, Brussels, Belgium; ^6^Biochimie et Biologie Moléculaire—LBMMS, Unité de diagnostic des pathologies dégénératives, Centre de Biologie et Pathologie Est, Groupement Hospitalier Est, Lyon, France; ^7^Univ. Lille, Inserm, CHU Lille, UMR-S-U1172, LiCEND, Lille Neuroscience & Cognition, LabEx DISTALZ, Lille, France; ^8^Laboratoire de biologie médicale de l’Institut de Neurologie de Tunis, Tunis, Tunisia; ^9^Laboratoire de Biologie, Institut Bergonié, Bordeaux, France; ^10^Cliniques universitaires Saint-Luc UCLouvain, Service de Neurologie, Brussels, Belgium; ^11^Hôpital Necker-Enfants malades, Paris, Laboratoire de Biochimie générale, DMU BioPhyGen, AP-HP.Centre—Université de Paris, Paris, France; ^12^Service de Biochimie, CHU Pellegrin, Bordeaux, France

**Keywords:** neurofilament, biomarkers, neurological and neurodegenerative diseases, cut-off, biological fluids

## Abstract

Neurological biomarkers are particularly valuable to clinicians as they can be used for diagnosis, prognosis, or response to treatment. This field of neurology has evolved considerably in recent years with the improvement of analytical methods, allowing the detection of biomarkers not only in cerebrospinal fluid (CSF) but also in less invasive fluids like blood. These advances greatly facilitate the repeated quantification of biomarkers, including at asymptomatic stages of the disease. Among the various informative biomarkers of neurological disorders, neurofilaments (NfL) have proven to be of particular interest in many contexts, such as neurodegenerative diseases, traumatic brain injury, multiple sclerosis, stroke, and cancer. Here we discuss these different pathologies and the potential value of NfL assay in the management of these patients, both for diagnosis and prognosis. We also describe the added value of NfL compared to other biomarkers currently used to monitor the diseases described in this review.

## Introduction

**Figure 1:**
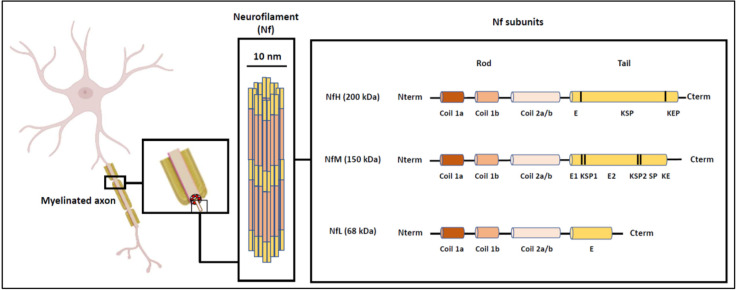
Structure and organization of neurofilaments (Nf), adapted from Gaetani et al. (2019).

Neurofilaments (Nf) belong to the family of intermediate filaments and their localization is exclusively neuronal. Nf differ from other types of intermediate filaments by the complexity of their structure and the composition of their subunits. Three subunits of Nf can be identified (and differentiated on SDS gel according to their molecular weight): NfH (heavy chain, 200 kDa), NfM (medium chain, 160 kDa), and NfL (light chain, 68 kDa; Yuan et al., 2017; Figure 1). Each protein subunit consists of a globular head, an alpha helix portion and a C-terminal domain of variable length, thus determining the molecular weight of each of these subunits (Yuan et al., 2006, 2012). Nf are involved in the radial growth of the axon during neuron development and in the maintenance of its structure and diameter, which are necessary for the transmission of nerve impulses. Nf are also involved in the organization and docking of the different components of the axon to the microtubule network.

**Figure 2:**
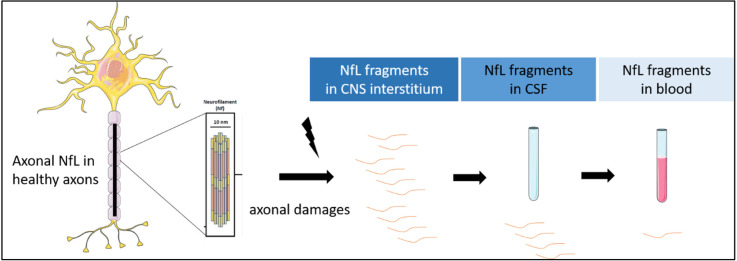
NfL release after axonal damages. NfL are detectable in CSF and blood.

Because of their enrichment in axons and their release into blood and cerebrospinal fluid (CSF) during neuronal damage, the measurement of NfL in these biological fluids as potential biomarkers of axonal damage, axonal loss and neuronal death raises many hopes in terms of diagnosis and prognosis (Figure 2).

NfL thus appears as one of the biomarkers of neurodegeneration, non-specific, but indirectly involved in the pathogenesis of many neurological diseases. These biomarkers are therefore being actively investigated and several assays for their quantification are currently available: the first one is based on an ELISA immunoassay (marketed by Uman Diagnostic, Umeå, Sweden), the second one is based on Simoa (single molecule array) technology and is implemented on the Quanterix^®^ (Billerica, MA, USA) Simoa^TM^ device, and the third one is an immunoassay, based on a microfluidic approach, which can be implemented on the Ella^TM^ device from the Protein Simple^®^ company. These three approaches use the same detection antibodies. A fourth approach, based on electro-chemiluminescence, can be implemented on the Mesoscale Discovery (MSD) devices (Rockville, MD, USA). However, the cut-offs for NfL measurement remain to be homogenized, or even defined, depending on the method, the clinical contexts and the physiological parameters influencing the concentrations such as age, body mass index or renal function (see Table 1). The objective of this article is to overview the interest of NfL in various neurodegenerative diseases and in other contexts of neurological impairment. We present the prognostic or diagnostic implications of measuring this biomarker in biological fluids. We also discuss the place and informative value of NfL in relation to other biomarkers commonly used to monitor the described pathologies.

**Table 1:** Diagnostic/prognostic blood NfL’s threshold in neurological pathological context, according to various technological approaches.

	Clinical contexts	sNfL cut-off value (pg/ml)	Technological approach used for cut-off definition	Reference
Neurodegenerative Diseases				
Alzheimer’s Disease (AD)	AD vs. control subjects	37.0	Simoa	Ashton et al. (2021) and Smirnov et al. (2022)
		19.3	Ella	Oeckl et al. (2022)
		26.6	MSD	Gaiottino et al. (2013)
	AD vs. FTD	42.7	Ella	Oeckl et al. (2022)
Parkinson’s syndrome (PS)	PS vs. control subjects	21.0	Simoa	Zhang et al. (2022)
	PD vs. atypical forms of parkinsonism (MSA, PSP)	14.8	Simoa	Marques et al. (2019)
	PD vs. atypical forms of parkinsonism (MSA, PSP, CBD)	17.2	Simoa	Quadalti et al. (2021)
	PD vs. PSP/CBD	16.6	Simoa	Quadalti et al. (2021)
	PD vs. MSA	17.2	Simoa	Quadalti et al. (2021)
	Atypical forms of parkinsonism vs. control subjects	13.6	Simoa	Marques et al. (2019)
Fronto-temporal dementia	Fronto-temporal dementia vs. PPD	19.5–33.0	Simoa	Forgrave et al. (2019)
Amyotrophic lateral sclerosis (ALS)	ALS vs. control subjects	500	Elisa	Shi et al. (2022)
	ALS vs. non-ALS patients	32.7	Simoa	Vacchiano et al. (2021)
Multiple sclerosis (MS)	OCB/Gd + occurence	31.0	Simoa	Bittner et al. (2020)
	Prognosis clinical disease activity	1.5 (z-score)	Simoa	Benkert et al. (2022)
Neurological Damages				
Traumatic brain injury (TBI)	TBI diagnosis	20.0	Simoa	Thelin et al. (2017)
	Prediction of intracranial lesions	8.38	Simoa	Kahouadji et al. (2022)
Stroke	Stroke prognosis	46.12	Simoa	Wang et al. (2021)
Oncology	Response to cancer therapy	28.0–60.0	Simoa	Schoeberl et al. (2022)
	Brain metastasis diagnosis	>22.0	Simoa	Kim et al. (2021)
	Chemotherapy toxicity	>36.0	Simoa	Huehnchen et al. (2022)
	ICANS occurence	75.0	Simoa	Schoeberl et al. (2022)

*AD, Alzheimer’s disease; PS, Parkinson’s syndromes; PD, Parkinson’s disease; FTD, fronto-temporal dementia; MSA, multisystem atrophy; PSP, progressive supranuclear palsy; CBD, corticobasal degeneration; PPD, primary psychiatric disorders; TBI, traumatic brain injury; MS, multiple sclerosis; ICANS, Immune Cell Associated Neurotoxicity Syndrome; OCB, oligoclonal bande in the CSF; Gd + , gadolinium lesions*.

## Nfl and Neurodegenerative Diseases

### Alzheimer’s disease

#### Background and state of the art

Alzheimer’s disease (AD) is the most prevalent cognitive neurodegenerative disease with approximately 900,000 people affected in France and more than 225,000 new cases per year (Helmer et al., 2006). From a pathophysiological point of view, AD is characterized by neurodegeneration due to the development of neurofibrillary tangles (NFTs, formed by aggregates of hyperphosphorylated tau protein) and amyloid plaques [formed by agglomerates of amyloid peptides Aβ produced by the cleavage of amyloid precursor protein (APP)]. The diagnosis of AD is based on clinical examination, interview with the patient and relatives, neuropsychological tests (e.g., mini-mental state examination, MMSE), imaging (structural Magnetic Resonance Imaging, MRI, FDG-PET, amyloid PET), and finally lumbar puncture with the quantification of CSF biomarkers Aβ42, Aβ40 (together with the ratio of Aβ42/Aβ40), total tau (t-tau), and phosphorylated tau on threonine 181 (p-tau). These CSF biomarkers perform well in the diagnosis of AD at the dementia stage (Hansson et al., 2006) but also allow diagnosis of the disease at early stage (MCI, mild cognitive impairment; Jack et al., 2013).

The number of publications related to the determination of NfL in AD is very high, both in CSF and in blood (546 referenced in Pubmed on 01/06/2022). As for almost all neurodegenerative diseases, NfL is increased in blood and CSF of AD patients (Gaetani et al., 2019; Mattsson et al., 2019; Delaby et al., 2020). Thus, AD patients can be differentiated from healthy controls with very good accuracy, by measuring NfL in both the CSF (Lista et al., 2017) and the blood (Forgrave et al., 2019; Mattsson et al., 2019). However, several studies reported absence of direct correlation between NfL concentrations in CSF or plasma and amyloid pathology (Aβ+ and Aβ−) as assessed by amyloid PET (Dhiman et al., 2020; Verberk et al., 2020). This suggests that changes in NfL concentration are independent of amyloid pathology in AD, whereas they are correlated with neurodegeneration and tauopathy (Dhiman et al., 2020).

#### Diagnostic and prognostic values

This almost systematic increase of NfL in neurodegenerative diseases limits the diagnostic interest of this biomarker in AD. However, it is noteworthy that the increase in NfL levels (both in blood and CSF) of AD patients remains more moderate than in most other neurodegenerative diseases (Gaetani et al., 2019). NfL may be differentially involved in the pathological processes of these disorders, in particular, neuronal death may be greater in some of them than in AD (which is chronic), thus generating higher levels of sNfL. In addition, pathologies associated with motor neuron death, such as ALS, might also exhibit higher levels of NfL than others.

Thus, for frontal forms of AD (e.g., primary progressive logopenic aphasia), the measurement of NfL could be of interest in the differential diagnosis with other primary progressive aphasias and, more generally, with frontotemporal lobar degeneration (FTLD), where NfL levels are higher (Pijnenburg et al., 2015; Disanto et al., 2016; Steinacker et al., 2017a; Paterson et al., 2018; Lleó A et al., 2019). The elevated levels of NfL in prion diseases would also allow a differential diagnosis between prions and AD (Steinacker et al., 2016; Thompson et al., 2018).

Changes in blood NfL appear to precede the first clinical manifestations of AD by about 16 years, as shown in longitudinal studies of AD mutation carriers (Preische et al., 2019): NfL could thus be used to monitor subjects with genetic risk factors for AD. Indeed, serum NfL has been shown to correlate with the estimated number of years before symptoms appear in carriers of autosomal dominant AD mutations (Sánchez-Valle et al., 2018) or in Down syndrome (Fortea et al., 2018, 2020).

Elevated CSF NfL is also associated with faster brain atrophy and cognitive decline in AD patients followed up longitudinally (Zetterberg et al., 2016; Osborn et al., 2019; Dhiman et al., 2020). Therefore, elevated CSF NfL in the early clinical stages of AD may predict accelerated cognitive decline and conversion to dementia in AD (Zetterberg et al., 2016; Lim et al., 2021). Thus, NfL assay could serve as a prognostic marker of worsening cognitive function in AD.

#### Position of NfL compared to other biomarkers and cut-off value

NfL measurement in CSF does not seem to provide further information to the amyloid biomarkers, t-tau and p-tau, already used to predict conversion to dementia in AD (Fortea et al., 2018).

Measurement of NfL in blood could be useful as a first line, i.e., screening test for AD and other neurodegenerative diseases. This would indeed be a straightforward implement due to the less invasive nature of blood collection compared to lumbar puncture.

Thresholds for the use of the blood NfL for diagnosis have been proposed in some studies, although the areas under the curve never reach 0.8. Two large studies found similar cut-off values with the Simoa technique, around 37 pg/ml: the first study being based on the comparison of amyloid+ vs. amyloid− subjects (defined on the basis of Aβ_1–42_ assays in CSF or amyloid PET imaging, *N* = 805; Ashton et al., 2021) and the second being based on a pathology cohort (*N* = 312) comparing Braak 0_II vs. Thal 4–5 and Braak V–VI subjects (Braak being the scale measuring DNF pathology and Thal amyloid pathology by brain region immunohistochemistry techniques; Smirnov et al., 2022). Using Ella technique, thresholds of 19.3 pg/ml and 42.7 pg/ml were reported to differentiate AD from controls and AD from FTD, respectively (Oeckl et al., 2022). When using the MSD technique, a threshold of 26.6 pg/ml was found to discriminate AD from controls (Gaiottino et al., 2013). These cut-offs are not comparable since the techniques are not standardized, but all studies agree in finding areas under the curve in the range of 0.7, which is insufficient for diagnostic use. But these blood thresholds might however be of interest for a first screening step. In addition, age-dependent cut-offs should increase the performance of the test. Finally, the combination of this test with blood measurements of amyloid and tau could also be interesting.

### Parkinson’s syndromes and synucleopathies

#### Background and state of the art

Parkinson’s disease (PD) is the second most common neurodegenerative disease in France (after AD) and the first leading cause of motor disorders. In France, 150,000 people are affected and 25,000 new cases are diagnosed every year (Elbaz et al., 2016). PD is due to the progressive loss of dopaminergic neurons of the nigro-striatal pathway in association with the formation of Lewy bodies corresponding to alpha-synuclein aggregation (Elbaz et al., 2016). Other neurodegenerative pathologies present alpha-synuclein aggregates, such as Lewy body disease (LBD), Parkinson’s dementia, multisystem atrophy (MSA) or idiopathic orthostatic hypotension (IOH). All these pathologies are part of the so-called synucleinopathies. The differential diagnosis between these synucleinopathies is complex and is compounded by diseases with atypical Parkinsonian syndromes, such as progressive supranuclear palsy (PSP) or corticobasal degeneration (CBD), making the differential diagnosis sometimes challenging. To date, no specific biomarkers can be used for such differential diagnosis.

The determination of NfL, both in CSF and in blood, has been intensively studied in the context of PD (156 results in Pubmed as of 01/06/2022). Interestingly, NfL levels do not seem to increase in the CSF of PD patients and several publications have reported similar levels to those of healthy controls (Gaetani et al., 2019).

#### Diagnostic and prognostic values

While NfL levels are not increased in either blood or CSF of parkinsonian patients, it is noteworthy that they rise in atypical forms of parkinsonian syndromes such as PSP, MSA, and CBD in both CSF (Bäckström et al., 2015; Herbert et al., 2015) and blood (Hansson et al., 2017; Lin et al., 2018; Marques et al., 2019; Ashton et al., 2021). This discrepancy could be due in part to milder and less extensive axonal degeneration in PD than in these atypical forms of parkinsonian syndromes. NfL levels are indeed related to severity, motor neurons scores, and stratification of PD.

Of note, while LBD patients (or those with parkinsonian dementia) also have significant elevated levels of NfL in CSF compared to healthy and PD patients, they have lower levels than PSP, MSA, and CBD patients (Hall et al., 2012). On the other hand, NfL levels in CSF are not very specific and do not differentiate LBD and AD patients (de Jong et al., 2007). The assessment of NfL in CSF or blood could thus be useful for the differential diagnosis of PD vs. atypical parkinsonian syndromes.

The prognostic value of NfL has been evaluated in PD and PSP, but no data are available on its prognostic value in MSA and CBD. In PD, baseline CSF NfL values are associated with mean change per year in *Dementia rating scale scores* (Olsson et al., 2019; Aamodt et al., 2021). The determination of NfL in CSF (Bäckström et al., 2015) and blood (Aamodt et al., 2021) thus predicts the risk of conversion to parkinsonian dementia in the following years, with not only cognitive but also motor impairment (Lerche et al., 2020; Mollenhauer et al., 2020). In PSP, higher baseline NfL values in CSF and blood appear to be associated with accelerated worsening of motor and cognitive symptoms (Rojas et al., 2016). Furthermore, some patients with HOI evolve to synucleinopathies with motor or cognitive impairment such as PD, LBD or MSA and the level of NfL in the CSF could help predict this conversion (Singer et al., 2021).

#### Position of NfL compared to other biomarkers and cut-off value

NfL assessed alone appears to have modest performance in predicting conversion from normal cognition to MCI or parkinsonian dementia individually, suggesting that NfL should be integrated into a multi-marker panel to improve prediction of clinical conversion to dementia. Some studies propose to incorporate, for example, Aβ_1–42_ assay in CSF for this purpose (Bäckström et al., 2015). Some articles have attempted to determine a threshold value using ROC curve to support the differential diagnosis of parkinsonian syndromes. Interestingly, all these studies achieved NfL quantification using the Simoa technique. Thus, a cut-off value of NfL at 21 pg/ml in plasma would allow a satisfactory discrimination of MSA patients and healthy subjects, with a sensitivity of 81% and a specificity of 93% (AUC = 0.912; Zhang et al., 2022). Similarly, NfL cut-off value in serum of 14.8 pg/ml (assessed by the Simoa approach) allows a clear discrimination (AUC = 0.91) between PD patients and those with atypical forms of parkinsonism (MSA and PSP), yielding a sensitivity of 86% and a specificity of 85% (Marques et al., 2019). Thus, among patients whose serum NfL concentration is above the cut-off value, the probability of having an atypical parkinsonism syndrome is 76% (positive predictive value), and patients whose serum NfL amount is below this cut-off value have a 92% probability of having PD (negative predictive value). In the same study, a cut-off value of 13.6 ng/L was used to differentiate patients with atypical parkinsonism from control subjects with a sensitivity of 93% and a specificity of 71% (AUC = 0.88; Marques et al., 2019). One study further detailed the differences in threshold values (assessed by Simoa) according to parkinsonian syndromes (Quadalti et al., 2021): for example, to differentiate PD from MSA, the best cut-off value for plasma NfL is at 17.2 pg/ml for a sensitivity of 90.3% and specificity of 96.4% (AUC = 0.972), and to differentiate a group of PD patients from a group of PSP/DCB patients, the optimal cut-off value is 16.6 pg/ml with a sensitivity of 88.7% and specificity of 87.8% (AUC = 0.936). When grouping atypical parkinsonian syndromes (MSA, PSP, DCB) vs. PD, the cut-off value of plasma NfL is 17.2 pg/ml for a sensitivity of 90.3% and a specificity of 91.7% (Quadalti et al., 2021). These results highlight the interest of NfL measurement in clinical setting, however, prospective validation and real-life clinical use are still needed to confirm such value.

### Fronto-temporal dementia (FTD)

#### Background and state of the art

Frontotemporal dementia (FTD) is a heterogeneous group of neurodegenerative diseases characterized by behavioral disorders and deficits in executive and language functions. It is the third most common cause of neurocognitive impairment after AD and LBD. In clinical practice, the challenge is to differentiate FTD patients with various primary psychiatric disorders (PPD), because of the overlap of some behavioral symptoms with FTD. To date, there is no validated biomarker to distinguish PPD from FTD but NfL, as a non-specific biomarker of neuronal death, appears to be promising to fill this gap in diagnosis.

Several studies have investigated NfL levels in FTD subjects: a total of 19 publications reporting NfL results in CSF (Goossens et al., 2018) and seven in serum were identified. Among these studies, three described NfL levels in patients with a definite diagnosis of FTD on *post-mortem* pathology analysis and 14 of them described this marker for an FTD population including also familial forms (Steinacker et al., 2018).

#### Diagnostic and prognostic values

The existing studies describe increased NfL concentrations in both matrices (CSF and serum) in FTD groups compared to PPD and control groups (Forgrave et al., 2019), with sensitivity and specificity values above 80%. The highest concentrations of NfL are observed in FTD associated with Amyotrophic Lateral Sclerosis (ALS, or Charcot disease; Pijnenburg et al., 2015). Thus, according to recently published international recommendations (Ducharme et al., 2020), NfL measurement in CSF or blood could be used in practice for the differential diagnosis between FTD and SPD, as long as validated thresholds are available.

Cut-off values found in studies comparing FTD vs. controls or FTD vs. PPD are similar (Davy et al., 2021), which suggests that NfL amounts are comparable in patients with PPD and healthy subjects; this is confirmed by studies comparing PPD and controls (Eratne et al., 2020).

Studies investigating the prognostic performance of NfL in the context of FTD described a 5-year survival estimate respectively at 36% in FTD patients with a high NfL concentration in the CSF (>3,675 pg/ml) and at 73% in patients whose NfL level was lower than 1,989 pg/ml (both measures determined by ELISA; Meeter et al., 2018). CSF NfL achieved an AUC of 0.87 (95% CI 0.81–0.92, *p* < 0.001), with a sensitivity of 79% and specificity of 89% (cutoff ≥1,613 pg/ml) to discriminate patients from controls (Meeter et al., 2018).

In a second study, high serum NfL concentrations were also associated with shorter patient survival and these concentrations were correlated with cortical atrophy of the prefrontal, temporal and parietal brain regions (Benussi et al., 2020). Interestingly, serum NfL concentrations showed a high accuracy in discriminating between FTD and healthy controls (area under the curve (AUC): 0.86, *p* < 0.001; Benussi et al., 2020). In subjects with genetic mutations in the *C9ORF72, GRN* or *MAPT* genes (pre-symptomatic subjects), increased serum NfL has been described during the conversion phase corresponding to the onset of symptoms (Wilke et al., 2022). Finally, as in ALS, serum NfL determination seems to be particularly relevant for monitoring future therapies in FTD (Toft et al., 2020; Saracino et al., 2021).

#### Position of NfL compared to other biomarkers and cut-off value

Low blood progranulin level is a validated biomarker used to predict the presence of GRN mutations for hereditary FTD (van Swieten and Heutink, 2008). Biomarkers are however lacking for the other etiologies and analysis of AD biomarkers in CSF (t-tau, p-tau, amyloid peptides) remains recommended to exclude this pathology as it is the main differential diagnosis for degenerative dementia (Ducharme et al., 2020). On the other hand, no difference in t-tau and p-tau concentrations was found between DFT and control groups (Abu-Rumeileh et al., 2018) and no difference in the concentration of other Nf subunits (NfM and NfH) is observed between FTD patients and PPD (Escal et al., 2022).

To date, no consensual NfL threshold value has been validated for the differential diagnosis between FTD and PPD. However, the studies mentioning cut-offs and using the UmanDiagnostics ELISA kit range from 1,063 pg/ml to 1,877 pg/ml in the CSF (Forgrave et al., 2019) and for the Simoa technique from 19.5 pg/ml to 33 pg/ml in blood (Forgrave et al., 2019). As the assay is not standardized, it is therefore necessary to assess and validate this threshold in each laboratory according to the technique used. This is not so easy, as it requires samples with a probable diagnosis, which is quite rare in this type of uncommon neurocognitive disorder.

### Other neurodegenerative proteinopathies

**Creutzfeld-Jakob disease (CJD)**, the most common form of prion disease, is responsible for extremely rapid cognitive decline. Its diagnosis is based on clinical criteria, EEG and the detection of the 14.3.3 protein in the CSF. Alternatively, a very large increase in t-tau concentration, with a high t-tau/p-tau ratio are strong arguments for the diagnosis. Recently, studies have shown the interest of NfL measurement in CSF (Abu-Rumeileh and Parchi, 2021) and in blood (Schmitz et al., 2022) to help in the early diagnosis of the disease and to differentiate it from other causes of dementia, in particular from progressive forms of AD. Of note, CSF NfL, either alone or in combination with other biomarkers, yielded a performance similar to t-tau in the distinction of prion disease from other neurodegenerative diseases (AUC 0.926 vs. 0.939) and showed even a higher diagnostic value than t-tau in the specific comparisons between atypical prion disease and other rapidly progressive neurodegenerative dementias (AUC 0.839 vs. 0.722; Abu-Rumeileh and Parchi, 2021). As for the blood, NfL shows lower values compared to blood t-tau for the discrimination between CJD and non-prion rapidly progressive dementias (AUC 0.497–0.724 and AUC 0.722–0.837, respectively; Abu-Rumeileh and Parchi, 2021).

**Huntington’s disease** is a rare, inherited disorder of the CNS. It is manifested by motor, cognitive, and psychiatric disorders. The mutated huntingtin (HTT) protein becomes abnormal and toxic to the neurons of the striatum when the number of CAG repeats is greater than 35. Mean concentrations of NfL in plasma at baseline were significantly higher in HTT mutation carriers than in controls [3.63 (SD 0.54) log pg/ml vs. 2.68 (0.52) log pg/ml, *p* < 0.0001] and the difference increased from one disease stage to the next, thus correlating with the severity of symptoms (Byrne et al., 2017, 2018; Rodrigues et al., 2020). Interestingly, the elevation of NfL precedes the clinic in children with the mutation (Byrne et al., 2018). Concentrations of NfL in CSF and plasma were correlated in mutation carriers (*r* = 0.868, *p* < 0.0001; Byrne et al., 2017).

### Amyotrophic lateral sclerosis

#### Background and state of the art

Amyotrophic lateral sclerosis (ALS) is a neurodegenerative disease of central and peripheral motor neurons, resulting in rapidly progressive amyotrophy and a greatly reduced life expectancy. Diagnosis can be challenging due to the heterogeneity of clinical presentations and the criterion of evolutivity. Therefore, it is common for patients initially identified as having ALS to have their diagnosis reconsidered as a slowly evolving motor neuron disease. In recent years, the NfL assay has emerged as a promising biomarker for the diagnosis and prognosis of ALS.

Published studies first focused on the determination of NfL in CSF (Zetterberg et al., 2007), then in blood (Gaiottino et al., 2013) but also on drying blood or plasma spots (Lombardi et al., 2020). To our knowledge, 36 articles illustrating the interest of NfL blood determination in the context of ALS have been published since 2013.

#### Diagnostic and prognostic values

Increased blood and/or CSF NfL levels have been reported in ALS patients (Xu et al., 2016; Verde et al., 2019), reflecting axonal damage of motor neurons during disease progression. The mean increase of NfL is significantly greater in ALS patients compared to patients with slowly progressing amyotrophy (Gaiani et al., 2017).

More recently, the interest of blood NfL in predicting the course of the disease has become apparent due to the correlation of this biomarker with the severity of clinical signs (Kojima et al., 2021) and/or the course of the disease (Thouvenot et al., 2020). Monitoring NfL blood levels could also allow the detection of pre-symptomatic forms of ALS in subjects at risk (Benatar et al., 2018). Finally, although no cure for ALS is yet available, NfL blood levels have been suggested as a monitoring marker for potential future therapies (Benatar et al., 2018; Witzel et al., 2021).

#### Position of NfL compared to other biomarkers and cut-off value

Diagnostic performance of blood NfL for ALS is superior to other biomarkers of axonal degeneration [such as S100B and progranulin (Steinacker et al., 2017b) or neuroinflammation (Brodovitch et al., 2021)]. This can be partly explained by the severity of the disease, but also by the fact that the degeneration affects large myelinated axons that have a strong expression of NfL. The combination of NfL and C-reactive protein in blood (De Schaepdryver et al., 2020) or ferroptosis markers (Devos et al., 2019) have been proposed for the prognostic evaluation of ALS. Determination of TDP-43 and t-tau proteins levels in CSF has also been described in this context (Kojima et al., 2021). A recent study described optimal cut-off values to discriminate between ALS and controls at 500 pg/ml (a sensitivity of 88.5% and specificity of 83.3%), using ELISA assay (Shi et al., 2022). When evaluating the ROC curves in discriminating patients with ALS and subjects with an alternative ALS-mimicking disease, sNfL (measured through Simoa) yields a diagnostic value of 0.873 ± 0.036 (sensitivity 84.7%, specificity 83.3%), for a cut-off 32.7 pg/ml (Vacchiano et al., 2021).

### Multiple sclerosis

#### Background and state of the art

Multiple sclerosis (MS) is a chronic inflammatory disease of the central nervous system (CNS), which can cause significant physical and cognitive disability and is responsible for a significant deterioration in quality of life. It is the most common neurological disease affecting young adults, three women for one man, with onset occurring mainly between the ages of 20 and 40. It is a multifactorial disease linked to genetic susceptibility factors in association with environmental risk factors and epigenetic factors leading to immune dysfunction (Reich et al., 2018). Eighty-five percent of multiple sclerosis evolves in the form of relapses alternating with phases of remission (so-called relapsing-remitting MS or RRMS). After 10–15 years of disease, RRMS may progress, especially without treatment, to a phase where disability progresses (so-called secondarily progressive MS or SPMS). Fifteen percent of patients present a progressive form from the start (so-called primary progressive MS or PPMS). Relapses are related to CNS invasion by pro-inflammatory peripheral immune cells causing multifocal demyelinating lesions and secondary axonal degeneration. Later phases of the disease are associated with diffuse microglial activation and the formation of ectopic lymphoid meningeal follicles (Cree et al., 2021). Although great progress in the accuracy of diagnostic criteria and immunotherapies for RR forms of MS has been achieved, no blood or CSF biomarkers to monitor disease activity or prognosis or finally to monitor response to treatment are available to date. NfL could be a useful biomarker in these indications, and more than 300 studies related to the measurement of NfL in MS have been published in CSF and since 2016 in serum (*n* = 178) or plasma (*n* = 49).

#### Diagnostic and prognostic values

Measurement of NfL is not relevant for the differential diagnosis of neuroinflammatory CNS pathology. However, various parameters related to the neuro-axonal damage induced by the inflammatory activity of MS are correlated with increased NfL levels in the CSF and in the blood: presence of a relapse (Barro et al., 2018), presence and number of gadolinium-enhancing lesions on gadolinium injection on MRI (Gd+), indicating “active” lesions (Novakova et al., 2017; Varhaug et al., 2018), increase in the number of new lesions on MRI (Bittner et al., 2020), and the brain volume (Barro et al., 2018). Interestingly, sNfL levels were described to be higher in RRMS than in clinically isolated syndrome patients (*p* = 0.001), thus suggesting sNfL might serve as a biomarker from very early stages of MS (Bittner et al., 2020). Importantly, the prediction accuracy of OCB (presence of oligoclonal bands in the CSF) and/or Gd+ (sensitivity: 72%, specificity: 76%, accuracy: 79%) were increased by including the 90th percentile of sNfL in addition to the above two variables (sensitivity: 73%; specificity: 79%, accuracy: 84%). These findings pointed towards a potential value of especially high sNfL levels (>31 pg/ml) at time of first demyelinating event as indicators of ongoing chronic CNS neuroinflammation and may be considered for inclusion in a future refinement of the McDonald criteria (Bittner et al., 2020). In the context of relapse occurrence or so-called “active” lesions, this increase may persist for a few weeks to a few months. Blood NfL levels also seem to correlate with markers of B cell activity in the CSF, such as the CD80 ^+^ marker or the CD20 ^+^/CD14 ^+^ ratio (Engel et al., 2019; Uher et al., 2021).

In the short term, elevation of NfL blood levels above the 80th percentile of measured samples correlates with worsening disability within 1 year, as measured by the Expanded Disability Status Scale (EDSS) score. This elevation is also predictive of the occurrence of relapses and progression of brain atrophy in the short term (Disanto et al., 2017; Barro et al., 2018; Calabresi et al., 2021a; Thebault et al., 2022). In the long term, elevated blood NfL levels seem to be predictive of increased brain atrophy at 5 or even 10 years (Kuhle et al., 2017; Barro et al., 2018; Chitnis et al., 2018; Cantó et al., 2019; Jakimovski et al., 2019; Srpova et al., 2021). Contrast enhancing and new/enlarging lesions were independently associated with increased serum neurofilament light chain (17.8% and 4.9% increase per lesion respectively; *P* < 0.001; Barro et al., 2018). The association with disability progression, however, is inconsistent across studies (Manouchehrinia et al., 2020a). One hypothesis that may explain the weaker long-term predictive value is that part of the increase in NfL blood levels related to chronic neurodegenerative processes (associated with the progression of disability in the long term) could be masked by neuro-axonal damage/injury due to the acute inflammatory activity of MS.

#### Blood NfL as a marker of treatment response

Numerous studies have reported decreased blood NfL levels following initiation of MS immunotherapies (Pop and Viuleţ, 1985; Disanto et al., 2017; Novakova et al., 2017; Varhaug et al., 2018; Cantó et al., 2019; Bittner et al., 2020; Calabresi et al., 2021a, b), so that this assay is currently included in most pharmaceutical clinical trials as a secondary measure of treatment effectiveness. The decrease appears to be greater upon initiation of high efficacy therapy (Delcoigne et al., 2020), compared to moderate or basic therapy (*p* < 0.05). This indicates that longitudinal sNfL changes rather than absolute sNfL values at a given time point might be indicative of disease activity and treatment stratification (Bittner et al., 2020). Longitudinal and individual measurement of NfL blood levels to assess the effect of initiated treatment on MS inflammatory activity is therefore promising. Early detection of a suboptimal response to treatment could indeed help to individualize therapeutic decisions, and some treatment strategies are already proposed on the basis of measured NfL values (Bittner et al., 2021; Thebault et al., 2021).

#### Position of NfL compared to other biomarkers and cut-off value

Astrocytic biomarkers [such as GFAP and chitinase-3-like protein 1 (CHI3L1)] are currently being studied to discriminate RRMS from SPMS patients (Selner, 1988; Huss et al., 2020). However their determination is not a substitute for other markers currently used in clinical routine (such as determination of the presence of CSF-specific IgG oligoclonal bands or visualization of lesion progression on MRI).

Comparison of ELISA and electrochemiluminescence techniques showed better sensitivity of the Simoa^TM^ platform for the determination of blood NfL in this context (Kuhle et al., 2016) and comparison between Simoa and Ella technologies showed similar results (Gauthier et al., 2021).

NfL levels increase with age (Gauthier et al., 2021) and fluctuate with body mass index and total blood volume (Manouchehrinia et al., 2020b), but are not influenced by gender (Harp et al., 2022). Renal function only influences NfL levels when the glomerular filtration rate is below 60 ml/min/1.73 m^2^ (Benkert et al., 2022). A meta-analysis in MS and independent cohort studies of young patients (between 18 and 50 years of age) did not show any association between CSF NfL level and age, in contrast to healthy subjects or those with other neurodegenerative diseases. This suggests that in younger patients, inflammatory activity may mask the effect of age on measured NfL levels (Bridel et al., 2019; Bittner et al., 2020; Manouchehrinia et al., 2020a; Rosso et al., 2021; Uher et al., 2021). Nevertheless, a recent study of more than 10,000 blood samples confirms that NfL level varies with age, with an increase of 2.5% per year, but that this increase is greater after 50 years of age (Benkert et al., 2022). This study enabled the determination and validation in independent cohorts of cut-off values expressed as percentile/Z-scores that quantify the deviation of serum NfL level from the control group, adjusted for age and body mass index: in this study, a sNfL Z score above 1.5 was associated with an increased risk of future clinical or MRI disease activity in all people with multiple sclerosis (odds ratio 3.15, 95% CI 2.35–4.23; *p* < 0.0001) and in people considered stable with no evidence of disease activity (2.66, 1.08–6.55; *p* = 0.034; Benkert et al., 2022). A freely available web application allows calculation of the adjusted percentile/Z-score from an individual NfL value^1^.

## NfL and Neurological Damages

### Traumatic brain injuries

#### Background and state of the art

Traumatic brain injury (TBI) is a major public health problem (more than 500 per day in France). About 80% of cases of TBI are classified as mild head injury (also called concussions). The morbidity associated with head trauma is considerable. Nearly 50% of victims have residual disabilities that may include progressive neurodegeneration, cognitive impairment, and dementia. Head injuries must be carefully diagnosed to foresee, anticipate, and treat the medical and/or social after-effects they cause. Blood and CSF biomarkers of neurodegeneration, particularly NfL, could help in the diagnosis and prognosis assessment.

#### Diagnostic and prognostic values

The number of publications regarding the interest of NfL in TBI is growing rapidly (391 articles in PubMed as of 01/06/2022). An increase in NfL levels in the CSF and blood is found during TBI, reflecting the axonal damage suffered (Khalil et al., 2018). Following TBI, whether severe or mild in athletes, NfL is detectable from the first hour and continues in a linear fashion with a maximum at Day 12 in the CSF and blood (Zetterberg et al., 2013; Zetterberg and Blennow, 2016; Shahim et al., 2017; McDonald et al., 2021).

The 24-h assessment of this biomarker could also be of prognostic interest. The NfL increase seems to be correlated with the severity of the damage found on the brain scan and with the severity of the neurological sequelae at 1 year, as well as with the survival of the patients (Shahim et al., 2016, 2018; Graham et al., 2021). Measurement at 24 h allows prediction of the long-term evolution of patients and high values are observed in case of unfavorable evolution. In athletes, NfL blood levels are correlated with the number of concussions and their impact (Verduyn et al., 2021). A recent study in rugby players showed that those who have suffered more than three concussions in a year maintained high NfL levels after the off-season, indicating chronic suffering. Chronically elevated levels of this biomarker have also been associated with the development of frontotemporal cognitive deficits and damage to the blood-brain barrier (Verduyn et al., 2021). Thus, a persistent increase in NfL could indicate the presence of progressive post-traumatic neurodegeneration. These results have yet to be confirmed by larger studies but suggest that NfL blood determination may be useful to identify patients at risk of developing chronic traumatic encephalopathy and to adapt their follow-up.

#### Position of NfL compared to other biomarkers and cut-off value

**Figure 3:**
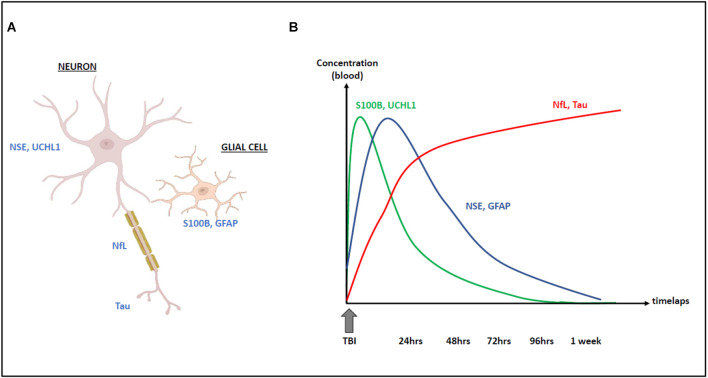
Cellular origin **(A)** and kinetics **(B)** of NfL increase following traumatic brain injury (TBI) compared with other biomarkers: S100B, UCH-L1, Tau, NSE, and GFAP.

The monitoring of serum biomarkers, such as S100B protein in TBI patients is of great interest for rapid and inexpensive diagnosis and prognostic evaluation. Other blood biomarkers are being studied and the NfL assay could be a complementary tool to enhance its diagnostic performance. Indeed, the cellular origin and the kinetics of these biomarkers are different (Figure 3): the determination of S100B must be performed within 3 h after the TBI [because of its half-life of 2 h (Jackson et al., 2000)], and the serum determination of NfL (whose half-life is longer and whose levels remain high several days after the TBI) would thus present a real informative added value in case of delayed patient management (Blomquist et al., 1997; Shahim et al., 2016; Thelin et al., 2017). A mean diagnostic serum threshold of 20 pg/ml is found in several studies in this context (Thelin et al., 2017). Other biomarkers of interest in this context are emerging, such as neuron-specific enolase (NSE), glial fibrillary acidic protein (GFAP), t-tau, and ubiquitin C-terminal hydrolase L1 (UCH-L1; Figure 3). Further studies comparing the diagnostic and prognostic performance of these markers are needed for their clinical use, alone or in combination, as well as for the advancement of CE and IVD labeling, which are essential for their use in clinical care. In the field of mild TBI (MTBI) and the interest of blood biomarkers in the reduction of unnecessary brain scans prescriptions, a recent study compared the diagnostic performances of S100B and NfL in the early management (blood samples within 3 h post-trauma) of 179 MTBI patients referred to an emergency department. S100B predicted intracranial lesions with a sensitivity of 100% and a 36% specificity. The NfL measurement did not enhance the predictive value. At a threshold of 8.38 ng/L, NfL predicted intracranial lesions with a 28% specificity. On the other hand, in the same population, NfL proved to be a high effective marker for the detection of patients with degenerative neurological pathologies, as shown by the data collected in this study (area under the ROC curve of 0.87 compared with only 0.57 for the S100B protein; Kahouadji et al., 2022).

Considering the extensive literature in this area, NfL is likely to be of greater interest for prognostic assessment. However, no cut-off value has yet been determined to predict the secondary occurrence of cognitive disorders.

### Stroke

#### Background and state of the art

Stroke is the worldwide leading cause of death and long-term morbidity. NfL may be of interest as a predictive biomarker for the outcome of ischemic stroke, and for the long-term consequences. Indeed, only one third of patients recover with minimal or no deficit, whereas the majority remain moderately or severely disabled for life. As life expectancy and population ages increase, stroke management has become a societal issue and efforts are underway to identify appropriate prognostic indicators for optimizing patient management.

#### Diagnostic and prognostic values

CSF and blood NfL levels are both increased after stroke, involving both small and large vessels, and several studies have investigated the relationship between this increase and the prognosis of ischemic stroke (Gattringer et al., 2017; Pujol-Calderón et al., 2019; Peters et al., 2020). A recent meta-analysis confirmed existence of a correlation between serum NfL level and the volume of the cerebral infarct area (Liu et al., 2020), with a pool adjusted odds ratios (ORs) from multivariate regression models, = 1.71 (95% CI: 1.17–4.29), representing that the patients with higher sNfL had a 1.71 times higher risk of poor functional outcome during follow-up (compared with lower sNfL patients; Liu et al., 2020). Kinetic assays reveal that the highest concentration would be reached 1 week after stroke (Tiedt et al., 2018; Gendron et al., 2020). Patients with the highest levels between 1 and 7 days after stroke were 1.7 times more likely to have sequelae within 3 months than those with low NfL levels. A large prospective study (*n* = 1,694 patients) recently confirmed that NfL levels at 48 h were an independent risk factor for cognitive sequelae within 3 months after stroke (Wang Z. et al., 2021), *p* < 0.001. Levels also correlated with the Rankin score used to measure disability secondary to stroke in the acute phase. Other studies show that initial NfL levels predict longer-term patient outcomes, particularly cognitive decline at 1 year (Wang J.- H. et al., 2021) and mortality (Gendron et al., 2020), regardless of whether the stroke was ischemic or hemorrhagic. The optimal cut-off value of the sNfL concentration was 46.12 pg/ml, which yielded a sensitivity of 71.0% and a specificity of 81.5%, with the area under the curve (AUC) at 0.785 (95% CI 0.762–0.808, *p* < 0.001; Wang Z. et al., 2021). These results demonstrate the value of early NfL determination as a prognostic biomarker.

CSF NfL levels are also increased in subarachnoid hemorrhage due to aneurysm rupture (Nylén et al., 2006; Gendron et al., 2020). As in stroke, the levels correlate with a poor prognosis, and in particular with 3-month mortality (Gendron et al., 2020; Hviid et al., 2020). The area under the receiver operating characteristic curve (ROC AUC) for discrimination of day-30 mortality was significant on admission [AUC = 0.83, 95% confidence interval (CI): 0.56–1.0] and increased on 24-h follow-up (AUC = 0.93, 95% CI: 0.84–1.0; Hviid et al., 2020).

NfL levels may remain elevated for up to 6 months after stroke. The sustained high levels could be explained by secondary Wallerian degeneration affecting motor neurons and by the poststroke inflammatory and immune response. High 6-month NfL levels correlate with the presence of secondary lesions and quantitative measure of secondary neurodegeneration detected by MRI (Tiedt et al., 2018; Peng et al., 2021).

#### Position of NfL compared to other biomarkers and cut-off value

Other biomarkers are studied in the stroke context: GFAP and Tau levels increase after stroke and are correlated with lesion volume and the NIHSS clinical score used for diagnosis and severity assessment. The kinetics of these biomarkers are different from NfL with a much faster rise and fall. The time between stroke and the determination of each of these biomarkers should be evaluated for optimal use (Pujol-Calderón et al., 2022). Although the place of blood NfL remains to be confirmed, it will undoubtedly improve, in combination with the available tools (biomarkers, clinical, and imaging scores), a better assessment of the prognosis of stroke patients. After an acute stroke, prophylaxis is implemented to prevent or reduce subsequent events, and a blood biomarker that captures subclinical events would help both monitor and determine the best treatment for this purpose (Campbell et al., 2019). Thus, blood levels of NfL are related to the risk of developing stroke in the years after the acute event and low NfL could be a real-time biomarker of the effectiveness of prophylactic treatment (Uphaus et al., 2019).

There is no validated consensus cut-off value for the assessment of stroke prognosis by blood NfL. Only one study proposes a threshold value of 46 pg/ml, determined by the Simoa approach, to identify patients who will remain with cognitive sequelae at 3 months [sensitivity of 71% and specificity of 81.5% (AUC 0.79; Wang Z. et al., 2021)]. This value should be adjusted according to the assay technique used, the patient’s history (neurodegenerative diseases) and age.

### Neurological damages in oncology

#### Background and state of the art

Neurological disorders in cancerology are numerous and varied in terms of both their etiology and the symptoms observed. They can be due to the primary cerebral localization of a tumor (glioblastoma, oligodendroglioma, medulloblastoma‥.), to the cerebral or medullary localization of metastases of an extra-cerebral cancer (lung, breast, melanoma, digestive cancer, lymphoma‥.), to a carcinomatous meningitis (inflammation by meningeal invasion by cancerous cells) or to autoimmune reactions triggered (paraneoplastic neurological syndrome). The treatment of these cancers is also responsible for neurological complications, and this neurotoxicity is in most cases dose-dependent. Neurological damage may affect the central nervous system (CNS), the peripheral nervous system (PNS) or both simultaneously. Early diagnosis of these disorders can often reduce symptoms and prevent permanent neurological deficits. Imaging tests such as MRI or electromyography (EMG) are used to identify these disorders, but there are no blood biomarkers (except for anti-neuronal antibodies) that can be useful for the diagnosis of these pathologies. Moreover, the currently available markers do not allow the evaluation of lesion progression and response to treatment. Various studies have explored the use of NfL in these indications.

#### Diagnostic and prognostic values

Primary CNS tumors and brain metastases are major causes of morbidity and mortality. The earlier the diagnosis, the better the management of the patients and the survival rates, which are anyway low in these cases. Several publications have thus focused on the use of NfL as a diagnostic and prognostic blood marker. High blood levels of NfL in brain metastases (Hepner et al., 2019; Kim et al., 2021; Lin et al., 2022) was reported, these levels being correlated with the number and size of metastases (Lin et al., 2022). A decrease in levels of NfL was also detected following treatment (Kim et al., 2021).

Interestingly, Winther-Larsen et al. demonstrated an increase in NfL in the blood (35 vs. 16 pg/ml, *p* = 0.001) early, even before the diagnosis of brain metastases (median 3 months before; Winther-Larsen et al., 2020). sNfL discriminated these patients with an area under the curve of 0.77 (0.66–0.89; Winther-Larsen et al., 2020). An increase in NfL could be measured median 3 months (range: 1–5) before the brain metastasis diagnosis. A very high level of NfL at the time of diagnosis seemed to be correlated with a lower survival, with an inferior survival [hazard ratio: 2.10 (95% confidence interval: 1.11–3.98; Winther-Larsen et al., 2020)]. Of note, these findings were recently confirmed in a cohort of lung cancer patients (Lin et al., 2022).

NfL determination may also have prognostic value in primary CNS tumors. Thus, Hepner et al. showed in a cohort of glioma patients that blood levels of NfL varied closely with tumor activity (Hepner et al., 2019), and that patients with progressive disease had on average 10-fold higher levels than those with stable disease.

#### Response to cancer therapy

Iatrogenic neurological complications can appear as early as the first days of treatment or even years later. Some alkylating agents are well known to be responsible for adverse effects with neurological manifestations (e.g., neuropathy and platinum salts). The increase in treatment lines and the development of new therapeutic modalities increase the risk of neurotoxicity. It can be challenging to make the diagnosis, but it is essential to get it done as soon as possible. Several studies have shown that the concentration of NfL correlates with the development and severity of toxicity resulting from various treatments. Thus, patients treated with paclitaxel, known to induce peripheral neurological manifestations, show an increase in blood NfL concentration 4 weeks after chemotherapy (compared to controls), resulting in an 86% sensitivity and 87% specificity (Huehnchen et al., 2022). An increase of sNFL of +36 pg/ml from baseline was associated with a predicted CIPN probability of more than 0.5 (Huehnchen et al., 2022).

The increase correlated with the development and severity of polyneuropathy (Huehnchen et al., 2022; Karteri et al., 2022). However, no association between increased blood NfL and cognitive impairment could be demonstrated in this context (Argyriou et al., 2022).

Emerging therapies such as antibody immunotherapy and CAR-T cells may also induce neurotoxicity, particularly autoimmune encephalitis, and the Immune Cell Associated Neurotoxicity Syndrome (ICANS), 1 week after treatment. In the case of post-immunotherapy autoimmune encephalitis, blood and CSF NfL rises and this result might be useful for tolerance monitoring (Piepgras et al., 2021). A very recent study reports that patients with ICANS after CAR-T therapy had elevated NfL levels (Schoeberl et al., 2022) and increased levels correlated with severity of the symptoms [(ICANS grade 0–1: 28.4 pg/ml (IQR, 19.2–49.7 pg/ml); ICANS grade 2–4: 60.0 pg/ml (IQR, 31.7–109.0 pg/ml); *p* < 0.01; Schoeberl et al., 2022)]. More surprisingly, patients who developed this neurotoxicity had already elevated pre-transplant levels of NfL (60 pg/ml vs. 28 pg/ml in the group that developed no or little neurotoxicity, as assessed by Simoa technology; Schoeberl et al., 2022).

These initial results are promising in terms of the usefulness of NfL in oncology but remain to be validated on larger and longer-term cohorts for neurotoxicity.

#### Position of NfL compared to other biomarkers and cut-off value

For metastases diagnosis, NfL threshold values may be defined according to age: for example, NfL levels >22 pg/ml (measured by the Simoa approach) in cancer patients aged 51–60 years may predict the presence of brain metastases (Kim et al., 2021). Other biomarkers are also being evaluated, in particular the GFAP protein which would, according to Darlix et al. (2021) outperform NfL as a diagnostic and prognostic factor for brain metastases in breast cancer patients. Further studies will determine whether the combination of the two biomarkers is worthwhile, but studies have already described increased levels of sNfL and GFAP in patients with CNS tumors with disease in progression vs. CNS with stable disease (*p* = 0.03 and *p* = 0.01, respectively; Hepner et al., 2019). Regarding chemotherapy toxicity, an increase in serum NfL concentration (assessed by Simoa) of more than 36 pg/ml (Huehnchen et al., 2022) or an elevated level at 3 or 4 weeks (>50 pg/ml and >85 pg/ml) is associated with high risk of developing polyneuropathy (Huehnchen et al., 2022; Karteri et al., 2022). In addition, high level of blood NfL could predict the occurrence of ICANS in case of CAR-T cell treatment (a threshold value of 75 pg/ml determined by the Simoa approach has been proposed; Schoeberl et al., 2022).

### Other NfL applications

It is difficult to be exhaustive on the use of NfL as this marker is in full expansion. For example, Nf could be of interest in other peripheral neuropathies, such as TTR amyloidosis (Ticau et al., 2021) and AL amyloidosis (Louwsma et al., 2021). Growing literature also highlights the predictive value of NfL concentrations in the intensive care setting, as it may be used to assess the risk of neurological events following either resuscitation (Fisse et al., 2021; Page et al., 2022) or cardiac arrest. Thus, high levels of blood NfL 48 h after cardiac arrest (>500 pg/ml) were described to predict neurological complications related to cerebral ischemia/hypoxia with high sensitivity (100%, 95%CI 70.0–100%) and specificity (91.7%, 95%CI 62.5–100%; Adler et al., 2022; Hoiland et al., 2022) and were correlated with EEG abnormalities, *p* < 0.001 (Grindegård et al., 2022).

Recent research has focused on the determination of NfL values in **children**, and norms could be proposed in the pediatric population (Nitz et al., 2021). Increased NfL is described to be correlated with the development of motor neurological disorders or retinopathy in premature infants (Goeral et al., 2021; Sjöbom et al., 2021). The value of NfL in CSF and blood also appears to be associated with the severity of inherited diseases with neurological impairment, such as SMA (Johannsen et al., 2021; Nitz et al., 2021), cerebral adrenoleukodystrophy (Wang et al., 2022) or mitochondrial diseases (Sofou et al., 2019). Finally, NfL could be relevant for monitoring treatment response (Ru et al., 2019).

## Conclusion

**Figure 4:**
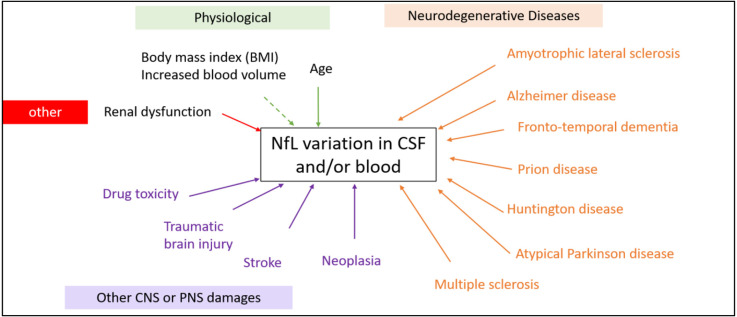
Physiological and pathological factors modifying the NfL levels in CSF and blood (non-exhaustive). A rise in NfL is not specific for a specific disease factor and may be caused by both neurodegenerative diseases or a head impact during sports for example.

Development of ultrasensitive immunoassays has made it possible to detect biomarkers of neuronal damage in easily collected blood samples, even though the concentrations are lower than in CSF. This opens a promising new avenue for the development of neurological biomarkers. Neurofilaments (Nf) are proteins selectively expressed in the cytoskeleton of neurons, the increase of which is a marker of neuronal damage. NfLs are highly specific to neurons but increase in many clinical settings and other candidates for neurological damage are currently under development. Indeed, neuronal damage is common and can be observed both during neurodegenerative diseases (such as AD, PD, FTD) but also in other neurological contexts (such as head injury, stroke or cancer). In this review, we have provided an overview of these different contexts, showing that the determination of NfL in biological fluids has a wide range of potential uses, sometimes for differential diagnosis, but more often for prognosis or monitoring of the therapeutic response of many neurological diseases (Figure 4). We discuss the place and informative added value of NfL compared to other commonly used biomarkers for monitoring neurodegenerative or no-neurodegenerative pathologies.

The NfL assay may be particularly relevant for the diagnosis of ALS or PD dementia for example. It would also be useful for the differential diagnosis between FTD and psychiatric disorders, between different Parkinsonian syndromes, or between Alzheimer’s disease and Prion disease, for which the levels of circulating NfL are very different. It is important to note that the level of NfL is a good marker of severity: its level is indeed correlated with a poor prognosis in many neurodegenerative diseases (ALS, Parkinson’s disease syndromes, MS, Huntington’s,‥.) and predicts the cognitive decline following repeated head trauma and stroke. The signs and symptoms on which the clinical diagnosis is based sometimes appear late in the course of the disease compared to the time of onset; the dynamics of blood NfL in the preclinical phases of neurological diseases and the possibility of detecting blood levels of NfL in an ultrasensitive manner could be used to identify diseases at an earlier stage. Finally, NfL can also be used to monitor the response to therapies and the neurotoxicity that such treatments may cause.

Nonetheless, it is important to keep in mind that NfL is a marker of neuroaxonal destruction independent of the mechanism causing the neuronal damage. The specific diagnosis will require consideration of a great deal of information, including the patient’s history, physical examination, imaging, and other laboratory tests. It seems essential to define reference ranges of normality for this biomarker, depending on the biological sample considered (CSF or blood), the analytical test performed, age and BMI for each medical indication in order to correctly interpret NfL results according to the context. Indeed, many factors other than the primary neurological disease, including age, BMI, cardiovascular risk factors, unrecognized head injury, etc., may be confounding factors that influence blood NfL and should therefore be taken into account when evaluating this biomarker in patients.

The homogenization of the thresholds of this biomarker according to the assay methods would allow a rapid development of its use in clinical routine for an optimized management of the patients. The extent of its use means that NfL could soon become a must for the clinical activity of the neurologist (Giovannoni, 2018; Lambertsen et al., 2020).

## Author Contributions

All authors contributed to the writing of the different parts of the article. SL, AB, and CD assembled the final version. All authors contributed to the article and approved the submitted version.

## Funding

No funding was required for this study which was carried out within the “Neurofilaments” working group of the French Society of Clinical Biology (SFBC).

## Conflict of Interest

The authors declare that the research was conducted in the absence of any commercial or financial relationships that could be construed as a potential conflict of interest.

## Publisher’s Note

All claims expressed in this article are solely those of the authors and do not necessarily represent those of their affiliated organizations, or those of the publisher, the editors and the reviewers. Any product that may be evaluated in this article, or claim that may be made by its manufacturer, is not guaranteed or endorsed by the publisher.

## Footnotes

^1^ https://shiny.dkfbasel.ch/baselnflreference

## Abbreviations

AD: Alzheimer’s disease
AUC: area under the curve
CBD: corticobasal degeneration
CJD: Creutzfeldt Jakob disease
CNS: central nervous system
CSF: cerebrospinal fluid
FTD: fronto-temporal dementia
Gd+: gadolinium lesions
GRN: progranulin
HTT: hungtintin
ICANS: Immune Cell Associated Neurotoxicity Syndrome
LBD: Lewy body disease
MCI: mild cognitive impairment
MS: multiple sclerosis
MSA: multisystem atrophy
NfL: neurofilament light
OCB: oligoclonal bande in the CSF
PD: Parkinson’s disease
PPD: primary psychiatric disorders
PS: Parkinson’s syndromes
PSP: progressive supranuclear palsy
ROC: receiver operating characteristic
TBI: traumatic brain injury.
